# Antibiogram Profiles and Risk Factors for Multidrug Resistance of *Salmonella enterica* Recovered from Village Chickens (*Gallus gallus domesticus* Linnaeus) and Other Environmental Sources in the Central and Southern Peninsular Malaysia

**DOI:** 10.3390/antibiotics9100701

**Published:** 2020-10-15

**Authors:** Saleh Mohammed Jajere, Latiffah Hassan, Zunita Zakaria, Jalila Abu, Saleha Abdul Aziz

**Affiliations:** Faculty of Veterinary Medicine, Universiti Putra Malaysia, Serdang, Selangor 43400 UPM, Malaysia; drmsjajere@unimaid.edu.ng (S.M.J.); zunita@upm.edu.my (Z.Z.); jalila@upm.edu.my (J.A.); saleha@upm.edu.my (S.A.A.)

**Keywords:** chicken, antibiotic, resistance, *Salmonella*, colistin, Malaysia

## Abstract

The emergence of multidrug resistance (MDR), including colistin resistance, among Enterobacteriaceae recovered from food animals poses a serious public health threat because of the potential transmission of these resistant variants to humans along the food chain. Village chickens or Ayam Kampung are free-range birds and are preferred by a growing number of consumers who consider these chickens to be organic and more wholesome. The current study investigates the antibiogram profiles of *Salmonella* isolates recovered from village chicken flocks in South-central Peninsular Malaysia. A total of 34 isolates belonging to eight serotypes isolated from village chickens were screened for resistance towards antimicrobials including colistin according to the WHO and OIE recommendations of critical antibiotics. *S.* Weltevreden accounted for 20.6% of total isolates, followed by serovars Typhimurium and Agona (17.6%). The majority of isolates (73.5%) demonstrated resistance to one or more antimicrobials. Eight isolates (23.5%) were resistant to ≥3 antimicrobial classes. Colistin resistance (minimum inhibitory concentrations: 4–16 mg/L) was detected among five isolates (14.7%), including *S.* Weltevreden, *S.* Albany, *S.* Typhimurium, and *Salmonella* spp. Univariable analysis of risk factors likely to influence the occurrence of MDR *Salmonella* revealed that the flock size, poultry production system, and use of antibiotics in the farm were not significantly (*p* > 0.05) associated with MDR *Salmonella*. The current study highlights that MDR *Salmonella* occur at a lower level in village chickens compared to that found in live commercial chickens. However, MDR remains a problem even among free-range chickens with minimal exposure to antibiotics.

## 1. Introduction

Increasing antibiotic resistance among foodborne pathogens is an emerging problem of global health importance [1] and the overuse of antibiotics in food animal production has been reported as one of its major drivers [2]. Colistin is a critically important antimicrobial in veterinary medicine [2], and is considered the drug of last resort against the emergent multidrug resistant (MDR) Gram-negative bacterial infections in humans, especially the carbapenem-resistant Enterobacteriaceae [3]. However, the overuse of colistin in the animal industry is reported to play an important role in the global emergence of colistin resistance [2]; as such, its use in livestock is being reconsidered in order to preserve drug efficacy [4,5]. Consequently an increasing number of consumers are choosing to consume meat from organically-grown sources or sourced from environments where there is less selective pressure that promotes the development of antibiotic resistance, thus reducing the transmission of resistant agents through food consumption [6,7]. Such environments typically include free-range production arrangements for food animals.

*Salmonella* is one of the most common foodborne bacteria worldwide [8]. *Salmonella* is a Gram-negative, rod-shaped bacilli and facultative anaerobe of the family Enterobacteriaceae, which can be broadly classified into two species based on their 16S rRNA sequence analysis: *Salmonella enterica* and *Salmonella bongori* [9]. There are more than 2500 serovars belonging to *S. enterica*, the majority of which are pathogenic and cause diseases in both animals and humans [10,11]. The global burden of non-typhoidal *Salmonella* (NTS) is increasing, with one study reporting approximately 94 million cases of NTS gastroenteritis, which is responsible for 155,000 deaths globally each year [8]. According to the study, the majority of the NTS burden is found in the Southeast Asian and Western Pacific region [8,12]. Of the 94 million cases reported, an estimated 80.3 million are thought to be of foodborne origin [13]. Poultry and poultry products (e.g., eggs and food products containing eggs) are commonly linked to NTS and have been demonstrated to serve as primary vehicles for human salmonellosis [8,14].

In Malaysia, several deaths and illnesses in recent years have been linked to foodborne NTS involving contaminated chicken and related products [15]. *Salmonella* contamination of various food products—sourced from either wet, retail markets or processing plants—such as chicken carcasses, chicken portions, various chicken organs (e.g., liver and gizzards), ready-to-eat foods, fruits, and vegetables, and other environmental sources, have been widely reported [16,17,18]. Only one of these studies was conducted on live birds in commercial poultry farms [19]. Nevertheless, no study to date has focused on village chickens in Malaysia.

Malaysian consumers increasingly prefer safer, wholesome, organic foods [20,21]. Moreover, in light of growing concerns over the transmission of antibiotic resistance via the food chain, the demand for these organic food products will likely increase over time. Therefore, village chicken production is an emerging niche market catering for this preference [22], with village chickens raised in a more “organic” free-range environment as compared to commercial broiler chickens. In this study, we described patterns of antibiotic resistance, including colistin resistance, and associated risk factors for the development of MDR in *Salmonella* isolates isolated from live local village chickens in South-central Peninsular Malaysia.

## 2. Results

### 2.1. Antimicrobial Resistance and Antibiogram Profiles of the Salmonella Isolates

Table 1 and Table 2 illustrate the isolate antibiotic resistance profile, multiple antibiotic resistance (MAR) index, and resistance phenotypes. Among the isolates, 26.5% (*n* = 9) were susceptible to all antibiotics, while 73.5% (*n* = 25) were resistant to at least one tested antibiotic. Multidrug resistance was displayed by eight isolates (23.5%). Ciprofloxacin (100%), gentamicin (97.1%), norfloxacin (97.1%), cefotaxime (97.1%), and ceftiofur (97.1%) were effective against most isolates. The highest level of resistance was observed for tetracycline (35.3%) and streptomycin (35.3%; Table 1).

**Figure 1:**
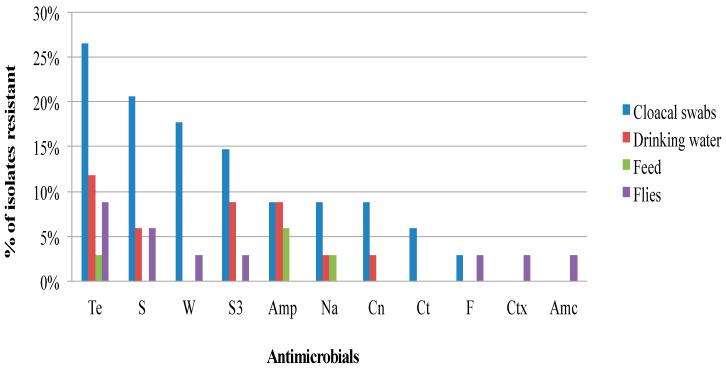
Frequency of *Salmonella* isolates recovered from various sources resistant to commonly used antibiotics from the South-central Peninsular Malaysia. (Te, Tetracycline; S, Streptomycin; W, Trimethoprim; S3, Sulfonamides; Amp, Ampicillin; Na, Nalidixic acid; Cn, Gentamicin; Ct, Chloramphenicol; F, Nitrofurantoin; Ctx, Cefotaxime; Amc, Amoxicillin-clavulanate).

**Figure 2:**
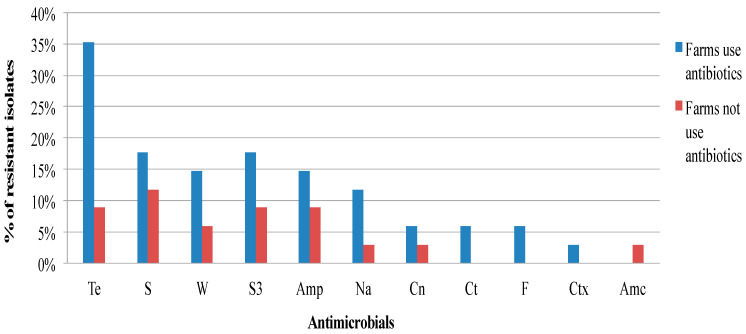
Frequency of resistance of *Salmonella* isolates recovered from village chickens of the South-central Peninsular Malaysia against antimicrobials according to the flocks with or without antibiotic use (Te, Tetracycline; S, Streptomycin; W, Trimethoprim; S3, Sulfonamides; Amp, Ampicillin; Na, Nalidixic acid; Cn, Gentamicin; Ct, Chloramphenicol; F, Nitrofurantoin; Ctx, Cefotaxime; Amc, Amoxicillin-clavulanate).

**Figure 3:**
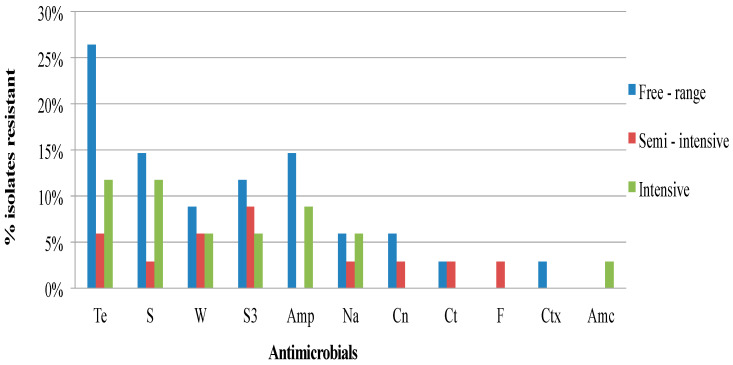
Frequency of resistance of *Salmonella* isolates from village chickens of the South-central Peninsular Malaysia according to the chicken production systems (Te, Tetracycline; S, Streptomycin; W, Trimethoprim; S3, Sulfonamides; Amp, Ampicillin; Na, Nalidixic acid; Cn, Gentamicin; Ct, Chloramphenicol; F, Nitrofurantoin; Ctx, Cefotaxime; Amc, Amoxicillin-clavulanate).

Figure 1, Figure 2 and Figure 3 show the frequency distribution of isolates resistant to commonly used antimicrobials according to the various sample sources, farms utilizing antimicrobials for treatment, and farm production systems. Generally, higher percentages of resistance were observed among isolates from farms that use antibiotics as compared to those farms that did not, and from the free-range system compared to the other systems. Most isolates found to manifest antimicrobial resistance were recovered from cloacal samples (Table 2). Table 3 presents the distribution and percentages of MDR *Salmonella* recovered from cloacal swabs, drinking water, and flies caught at the farm.

For colistin, the minimum inhibitory concentrations (MICs) of the *Salmonella* isolates ranged from 0.25 to 16 mg/L (Table 2). Five (14.7%) of the *Salmonella* isolates had MICs of 4–16 mg/L. According to the European Committee on Antimicrobial Susceptibility Testing (EUCAST) MIC breakpoints for colistin, MIC 4 mg/L is considered resistant to colistin (http://www.eucast.org/clinical-breakpoints/). These isolates comprise of *S.* Weltevreden, *S.* Typhimurium, *S.* Albany, and *Salmonella* spp. (Table 2). The two former serovars originated from free range (*n* = 3), while the latter two were from semi-intensive (*n* = 2) production systems. All but *S*. Typhimurium were MDR (Table 2).

### 2.2. Analysis of Risk Factors for MDR Salmonella

Simple logistic regression analysis of the risks factors associated with the development of MDR *Salmonella* revealed that none of the factors investigated (including flock size, poultry production system, and use of antibiotics) were not significantly associated (*p* > 0.05) with MDR *Salmonella* (Table 4).

## 3. Discussion

Food safety issues, such as the presence of residual chemicals and antibiotic resistance, have resulted in an increased willingness among consumers to pay more for organically raised foods. Many Malaysians consume raw village chicken eggs because they believe it is medicinal, nutritious, safe, and antibiotic-free [6]. *Salmonella* isolates found in this study were susceptible to most of the antibiotics tested. High sensitivity levels were demonstrated against gentamicin, ciprofloxacin, cefotaxime, norfloxacin, and ceftiofur. This finding is consistent with similar studies that report a minimum level of resistance against the aforementioned antibiotics from local commercial chickens and chicken carcasses [23,24,25,26]. Complete susceptibility against cefotaxime, ciprofloxacin, and gentamicin was also demonstrated by *Salmonella* isolates recovered from Spanish broiler flocks [27]. In the present study, we found the highest levels of resistance were against tetracyclines (35.3%), streptomycin (35.3%), sulfonamides (29.4%), and trimethoprim (20.6%). In contrast, these levels were much lower than those reported against the same antibiotics (34–100%) for *Salmonella* isolates originating from commercial chickens in Malaysia [24,26]. The highest levels of resistance manifested by the isolates from the aforementioned studies were against tetracycline (100%), ampicillin (100%), clindamycin (100%), and ciprofloxacin (83%). Moreover our *Salmonella* isolates were susceptible to ciprofloxacin in contrast to that reported elsewhere as being between 30.8% and 96% [28,29,30,31].

Other studies around the world have shown the presence of antimicrobial resistant *Salmonella* recovered from backyard or free-range chickens. In most of these studies, resistance was demonstrated against ampicillins and tetracyclines [32,33,34,35,36]. Eight (23.5%) of the *Salmonella* isolates in this study were resistant to three or more antimicrobial agents (MDR; Table 3). This is comparatively lower than those reported in previous studies in commercial chickens in Malaysia, where a 100% and 75% of *Salmonella* recovered from cloacal swabs and chicken carcasses/products [26] respectively exhibited MDR [37]. In China, a similar level of MDR (26.3%) of *Salmonella* was also reported among free-range chickens [35].

Elsewhere, in commercial poultry as well as in retail chicken meats, or other related products, reports of the frequency of MDR *Salmonella* vary widely. For example, MDR *Salmonella* has been found in 80% of chicken carcasses and other related products in Egypt [28], with a similar level found in retail chicken meats in China [29]. Conversely, Spanish broiler flocks [27], as well as poultry houses [38] and broiler chicken farms [39] in Brazil reported MDR *Salmonella* as lower than 20%. MDR has been well documented among epidemiologically important serovars, such as *S.* Typhimurium, which can exhibit resistance to up to 13 antibiotics [25], and *S.* Enteritidis, which can demonstrate resistance to up to six antibiotics [24]. In our study, isolates of the aforementioned serotypes were not MDR (Table 3). In Iran, a similar study found that *S.* Typhimurium isolates recovered from backyard chickens exhibited resistance against tetracycline, sulfamethoxazole, and trimethoprim [36].

The level of MDR found in this study, although meaningfully lower than those reported in commercial chickens locally and elsewhere, is worrying given that antibiotics are not routinely used in the production of village chickens. However, recent reports of the discovery of antibiotic resistance among animals in geographically remote locations where commercial antibiotics have not been applied may help to explain our findings [40]. The practice of free-range farming, allowing birds to roam freely, may result in their exposure to natural environmental hazards, such as untreated water and soil, which have been well documented to harbor drug-resistant foodborne pathogens [41,42,43,44]. Recent reports suggest that wild birds and gulls, creatures not exposed to the selective pressure of antibiotic use, have also been found to harbor MDR organisms in light of their continuous exposure to the natural environment [45,46,47,48]. It is also possible that feeding village chicken table scraps, which may contain resistant bacteria or materials, has additionally contributed to our observation of the phenomena. We found isolated MDR *Salmonella* from the cloacal swabs, drinking water and flies (Table 3). These findings further support the role of the environment in the dissemination and recirculation of MDR *Salmonella* [49,50]. Shang et al. (2018) found a significantly higher isolation rate of MDR *Salmonella* from litter samples compared to other samples in broiler farms, indicating the significance of poultry litters in the in-door environmental transmission of MDR *Salmonella* [31].

Five (14.7%) of the *Salmonella* isolates were resistant to colistin, with MICs in the range of 4–16 mg/L (Table 2). All of these isolates originated from free-range and semi-intensive production systems where chickens spend much of their time free grazing. Since the first report of the plasmid-mediated colistin resistance gene (*mcr-1*) from China in 2015, reports on resistance have emerged from more than 30 countries across several continents. These resistant isolates were recovered from several sources, such as environmental samples, food-producing animals, ready-to-eat foods, fruits, and vegetables, and humans [51,52,53,54,55,56,57,58,59,60]. Colistin resistance has been reported worldwide [58]; nevertheless, the burden of colistin resistance is highest in Asian countries. For example, several reports have documented the identification of MCR-1-producing *E. coli* isolates from samples of chickens and chicken meat, pigs and piglets, cattle, calves, turkeys, and humans in Cambodia [61,62], South Korea and China [53,63,64], Japan [65,66], Laos [67], Nepal [68], Pakistan [69,70], India [71], Thailand [67,72], and Vietnam [73,74]. In 2015, a study in Laos reported a possible clonal transmission of colistin-resistant *E. coli* between a domesticated pig and a human [67]. Although the direction of the transmission cannot be ascertained, the findings indicate that the fluidity of resistant agent transmission between species is of animal and public health significance.

## 4. Materials and Methods

### 4.1. Source of the Isolates

The design of this study, sampling, and data collection, and the process of isolation and identification have been described in our previous publication [75]. Briefly, isolates and data were obtained from a cross sectional study involving 35 village chicken farms across 4 states in central and Southern Peninsular Malaysia. Table 5 shows the distribution of *Salmonella* serotypes analyzed in this study. All work was carried out at the Veterinary Public Health Laboratory, Faculty of Veterinary Medicine, Universiti Putra Malaysia.

### 4.2. Antimicrobial Susceptibility Testing

Antibiotic sensitivity testing of the isolates against 15 antibiotics was performed using the agar disc diffusion method [76] using antibiotic discs; ampicillin (10 μg), nalidixic acid (30 μg), chloramphenicol (30 μg), ciprofloxacin (5 μg), gentamicin (10 μg), nitrofurantoin (300 μg), trimethoprim (5 μg), tetracycline (30 μg), kanamycin (30 μg), amoxicillin–clavulanate (20/10 μg), cefotaxime (30 μg), norfloxacin (10 μg), sulfonamides (300 μg), streptomycin (10 μg), and ceftiofur (30 μg). For colistin, the minimum inhibition concentration were determined by broth microdilution, using the MIC-Strip Colistin (MERLIN Diagnostika GmbH, Bornheim, Germany), in accordance with the international standard reference method (ISO 20776-1), and as recommended by the EUCAST subcommittee [77]. Test procedures were performed according to the manufacturer’s instructions, and interpretative MIC breakpoints were based on the EUCAST criteria (http://www.eucast.org/clinical-breakpoints/).

Antibiotics were selected based upon the recommendations of the World Health Organization (WHO) and World Organization for Animal Health for the use of antimicrobials in both human and food-producing animals. Colistin was recently added to the list of critically important antibiotics used in food-producing animals [2,78].

The diameter of the zone of inhibition (mm) were interpreted according to the criteria of the The Clinical and Laboratory Standards Institute (CLSI) [76]. Strains were subsequently evaluated according to the CLSI breakpoints as susceptible, intermediate, or resistant. Digital Vernier calipers were used to measure the diameter of the zones of inhibition. *S.* Typhimurium (ATCC 29213) and *S.* Enteritidis (ATCC 25922) were used as reference strains for antibiotic disc control. A resistant isolate was defined as an isolate resistant to one or more of the agents tested, whereas isolates resistant to three or more classes of antimicrobials were classified as multidrug resistant (MDR) [79]. The multiple antibiotic resistance (MAR) index was defined as the proportion formed by the number of antibiotic types to which a particular isolate displayed resistance, to the total number of antibiotics to which the isolate had been evaluated for susceptibility [80,81]. MAR is a good tool for assessing health risk, and is used to determine whether an isolate originates from a region of high or low antibiotic usage [81]. An MAR index of >0.2 reflects a bacteria from a high risk source of contamination where several antibiotics or growth promoters are used, whereas <0.2 represent those from a low risk source or source with less antibiotic use [80,81].

MAR index = *a/b*, where “*a*” is the number of antibiotics to which the isolates were resistant, and “*b*” is the total number of antibiotics to which the isolate was exposed [80,81].

### 4.3. Data Analysis

Data generated from the study was subjected to descriptive analysis using MS Excel (version 2011) to obtain percentages and proportions. SPSS (version 22.0, IBM, Armonk, NY: IBM Corp.) was used for all analyses. Chi-square, Fisher’s exact test, or simple logistic regression was used for the univariable exploratory analysis to identify risk factors associated with the outcome variable. The outcome variable was the presence or absence of multidrug resistant isolates (*Salmonella* isolates manifesting resistance to ≥3 classes of antimicrobial agents). The statistical significance level was set at α = 0.05.

## 5. Conclusions

The present study found that the level of resistance to antibiotics among isolates recovered from local village chickens was much lower than those found in commercial chickens and their products. Therefore, village chickens could be an alternative to those consumers seeking to reduce exposure to antibiotic resistant pathogens via the food chain. The findings also highlight the presence of MDR isolates, including those resistant to colistin, despite minimal antibiotic usage in free-range chicken production systems. We conclude that prolonged exposure to the natural environment plays a critical role in the transmission of resistance. Nevertheless, the role of the environment in the perpetuation and transmission of agents of resistance requires further study in light of emerging global trends in free-range food animal production.

## Figures and Tables

**Table 1:** Antibiogram of *Salmonella* isolates recovered from village chickens of the central and Southern Peninsular Malaysia.

Antimicrobial Agents	No. Tested	Antibiogram of *Salmonella* Isolates		
		Resistant (%)	Intermediate (%)	Sensitive (%)
Gentamicin	34	00	1 (2.9)	33 (97.1)
Amoxicillin-Clavulanate	34	2 (5.9)	2 (5.9)	30 (88.2)
Nitrofurantoin	34	4 (11.8)	00	30 (83.8)
Ciprofloxacin	34	00	00	34 (100)
Kanamycin	34	1 (2.9)	2 (5.9)	31 (91.2)
Trimethoprim	34	7 (20.6)	00	27 (79.4)
Norfloxacin	34	00	1 (2.9)	33 (97.1)
Tetracycline	34	12 (35.3)	1 (2.9)	21 (61.8)
Nalidixic acid	34	5 (14.7)	00	29 (85.3)
Chloramphenicol	34	4 (11.8)	00	30 (88.2)
Ampicillin	34	6 (17.6)	00	28 (82.4)
Cefotaxime	34	1 (2.9)	00	33 (97.1)
Streptomycin	34	12 (35.3)	11 (32.4)	11 (32.4)
Sulfonamides	34	10 (29.4)	00	24 (70.6)
Ceftiofur	34	0	1 (2.9)	33 (97.1)
Colistin ^1^	34	5 (14.7)	0	29 (85.3)

^1^ Minimum inhibitory concentrations (MICs) was determined by the microbroth dilution method using the MIC-Strip kit (MERLIN Diagnostika GmbH, Bornheim, Germany).

**Table 2:** Antibiotic resistance patterns of *Salmonella* recovered from village chickens in the South-central Peninsular Malaysia.

Sources	Resistance Profiles	*Salmonella* Serovars	Colistin MIC ^1^		MAR Index ^2^
			Conc. (mg/L)	R/I/S ^3^	
Cloacal swab	AmpTeWS_3_SFNaCt	*Salmonella* spp.	4	R	≥0.2
Flies	TeWS_3_SFCtxAmc	*S.* Molade	2	S	≥0.2
Cloacal swab	AmpS_3_WNaCn	*S.* Weltevreden	8	R	≥0.2
Cloacal swab	AmpS_3_WNaCn	*S.* Weltevreden	8	R	≥0.2
Drinking water	AmpTeS_3_SCn	*S.* Albany	16	R	≥0.2
Cloacal swab	TeWS_3_	*S.* Corvallis	0.25	S	≥0.2
Cloacal swab	TeS_3_S	*Salmonella* spp.	2	S	≥0.2
Drinking water	TeS_3_S	*S.* Albany	2	S	≥0.2
Feed	TeNa	*S.* Weltevreden	2	S	<0.2
Drinking water	TeNa	*Salmonella* spp.	2	S	<0.2
Drinking water	AmpTe	*S.* Weltevreden	2	S	<0.2
Drinking water	AmpS_3_	*S.* Weltevreden	0.5	S	<0.2
Cloacal swab	TeS	*S.* Molade	0.5	S	<0.2
Cloacal swab	TeW	*S.* Typhimurium	4	R	<0.2
Cloacal swab	TeW	*S.* Agona	2	S	<0.2
Cloacal swab	Ct	*S.* Enteritidis	2	S	<0.2
Cloacal swab	Te	*Salmonella* spp.	0.25	S	<0.2
Cloacal swab	Te	*S.* Typhimurium	0.25	S	<0.2
Cloacal swab	Te	*S.* Typhimurium	0.25	S	<0.2
Feed	Amp	*S.* Typhimurium	0.5	S	<0.2
Feed	Amp	*S.* Albany	0.25	S	<0.2
Cloacal swab	S	*S.* Agona	0.25	S	<0.2
Cloacal swab	S	*S.* Agona	0.25	S	<0.2
Cloacal swab	S	*S.* Agona	0.25	S	<0.2
Cloacal swab	S	*S.* Agona	0.25	S	<0.2

^1^ MIC, minimum inhibitory concentration (mg/L) according to the MIC-strip microbroth dilution method, ^2^ MAR, multiple antibiotic resistance index, MAR index = No. of resistance antibiotic types/total number of antibiotic types tested, ^3^ R/I/S, Resistant, Intermediate or Susceptible according to the European Committee on Antimicrobial Susceptibility Testing (EUCAST) guidelines (http://www.eucast.org/clinical-breakpoints/) (Te, Tetracyclines; Amp, Ampicillin; S, Streptomycin; W, Trimethoprim; S_3_, Sulfonamides; Amc, Amoxicillin-clavulanate; Na, Nalidixic acid; Ct, Chloramphenicol; Ctx, Cefotaxime; Cn, Gentamicin; F, Nitrofurantoin; K, Kanamycin).

**Table 3:** Distribution of multidrug resistant (MDR) *Salmonella* isolates recovered from native chickens of the South-central Peninsular Malaysia.

Sources	No. of MDR *Salmonella*	% Positive ^1^
Cloacal swabs (*n* = 17)	5	29.4%
Drinking water (*n* = 9)	2	22.2%
Flies (*n* = 3)	1	33.3%

^1^ Fisher’s exact test, *p* > 0.05 (not significant).

**Table 4:** Risk factors associated with the occurrence of MDR *Salmonella* amongst village chickens from the South-central Peninsular Malaysia.

Variables	Frequency	MDR (%)	OR (95% CI) ^1^	*p*-Value
Flock size (number of birds)				
<500	22	6 (27.3)	1.84 (0.32–15.52)	0.533
≥500	12	2 (16.7)	Reference	-
Poultry production system				
Free-range	22	5 (22.7)	0.78 (0.14, 4.90)	0.779
Semi-intensive/Intensive	12	3 (25.0)	Reference	-
Use of antibiotics in the farm				
Yes	20	6 (30.0)	1.96 (0.34–16.20)	0.478
No	14	2 (14.3)	Reference	

^1^ OR, Odds ratio, CI, Confidence interval.

**Table 5:** Distribution of *Salmonella* serovars isolated across different samples from village chicken flocks in the South-central Peninsular Malaysia.

Serovars Isolated	Sources	Number (%) Total *n* = 34
*Salmonella* Weltevreden	Cloacal swabs, feed, water	7 (20.6)
*Salmonella* Typhimurium	Cloacal swabs, feeds	6 (17.6)
*Salmonella* Agona	Cloacal swabs	6 (17.6)
*Salmonella* Enteritidis	Cloacal swabs, water, feeds	3 (8.8)
*Salmonella* Albany	Water, feeds	3 (8.8)
*Salmonella* Molade	Cloacal swabs, flies	2 (5.9)
*Salmonella* Corvallis	Cloacal swabs	2 (5.9)
*Salmonella* Schleissheim	Flies	1 (2.9)
*Salmonella* spp.	Cloacal swabs, water	4 (11.8)

## References

[1] Brown A.C., Grass J.E., Richardson L.C., Nisler A.L., Bicknese A.S., Gould L.H. (2016). Antimicrobial resistance in Salmonella that caused foodborne disease outbreaks: United States, 2003–2012. Epidemiol. Infect..

[2] Collignon P.C., Conly J.M., Andremont A., McEwen S.A., Aidara-Kane A. (2016). World Health Organization Ranking of Antimicrobials According to Their Importance in Human Medicine: A Critical Step for Developing Risk Management Strategies to Control Antimicrobial Resistance from Food Animal Production. Clin. Infect. Dis..

[3] Li J., Nation R.L., Turnidge J.D., Milne R.W., Coulthard K., Rayner C.R., Paterson D.L. (2006). Colistin: The re-emerging antibiotic for multidrug-resistant Gram-negative bacterial infections. Lancet Infect. Dis..

[4] Kempf I., Jouy E., Chauvin C. (2016). Colistin use and colistin resistance in bacteria from animals. Int. J. Antimicrob. Agents.

[5] Poirel L., Jayol A., Nordmann P. (2017). Polymyxins: Antibacterial Activity, Susceptibility Testing, and Resistance Mechanisms Encoded by Plasmids or Chromosomes. Clin. Microbiol. Rev..

[6] Hassan L., Zaleha Suhaimi S., Saleha A.A. (2005). The detection and comparison of antimicrobial resistance pattern of vancomycin-resistant enterococci and Salmonella isolated from eggs of commercial layers and free-range chickens. J. Vet. Malays..

[7] Miao Z.H., Glatz P.C., Ru Y.J. (2005). Free-range Poultry Production—A Review. Asian Australas. J. Anim. Sci..

[8] Majowicz S.E., Musto J., Scallan E., Angulo F.J., Kirk M., O’Brien S.J., Jones T.F., Fazil A., Hoekstra R.M., The International Collaboration on Enteric Disease “Burden of Illness” Studies (2010). The Global Burden of Nontyphoidal Salmonella Gastroenteritis. Clin. Infect. Dis..

[9] Eng S.-K., Pusparajah P., Ab Mutalib N.-S., Ser H.-L., Chan K.-G., Lee L.-H. (2015). Salmonella: A review on pathogenesis, epidemiology and antibiotic resistance. Front. Life Sci..

[10] Shah D.H., Paul N.C., Sischo W.C., Crespo R., Guard-Petter J. (2017). Population dynamics and antimicrobial resistance of the most prevalent poultry-associated Salmonella serotypes. Poult. Sci..

[11] Mezal E.H., Sabol A., Khan M.A., Ali N., Stefanova R., Khan A.A. (2014). Isolation and molecular characterization of Salmonella enterica serovar Enteritidis from poultry house and clinical samples during 2010. Food Microbiol..

[12] Prestinaci F., Pezzotti P., Pantosti A. (2015). Antimicrobial resistance: A global multifaceted phenomenon. Pathog. Glob. Heal..

[13] Mouttotou N., Ahmad S., Kamran Z., Koutoulis K. (2017). Prevalence, Risks and Antibiotic Resistance of Salmonella in Poultry Production Chain. Current Topics in Salmonella and Salmonellosis.

[14] Foley S.L., Lynne A.M., Nayak R. (2008). Salmonella challenges: Prevalence in swine and poultry and potential pathogenicity of such isolates1,2. J. Anim. Sci..

[15] New C., Ubong A., Premarathne J., Thung T., Lee E., Chang W., Loo Y., Kwan S., Tan C., Kuan C. (2017). Microbiological food safety in Malaysia from the academician’s perspective. Food Res..

[16] Rusul G., Khair J., Radu S., Cheah Y.K., Yassin R. (1996). Prevalence of Salmonella in broilers at retail outlets, processing plants and farms in Malaysia. Int. J. Food Microbiol..

[17] Adzitey F., Rusul G., Huda N. (2012). Prevalence and antibiotic resistance of Salmonella serovars in ducks, duck rearing and processing environments in Penang, Malaysia. Food Res. Int..

[18] Arumugaswamy R., Rusul G., Hamid S.A., Cheah C. (1995). Prevalence of Salmonella in raw and cooked foods in Malaysia. Food Microbiol..

[19] Ong L.P., Muniandy K., How S.P., Yip L.S., Lim B.K. (2014). Salmonella Isolation from Poultry farms in Malaysia from 2011 to 2013. Malays. J. Vet. Res..

[20] Somasundram C., Razali Z., Santhirasegaram V. (2016). A Review on Organic Food Production in Malaysia. Horticulturae.

[21] Ishida A., Law S.-H., Aita Y. (2003). Changes in food consumption expenditure in Malaysia. Agribusiness.

[22] Rahman W.A., Haziqah F. (2015). Ectoparasitic fauna of scavenging chickens (Gallus domesticus) from Penang Island, Peninsular Malaysia. Malays. J. Vet. Res..

[23] Cardoso M.O., Ribeiro A.R., Dos Santos L.R., Pilotto F., De Moraes H.L., Salle C.T.P., Rocha S.L.D.S., Nascimento V.P.D. (2006). Antibiotic resistance in Salmonella Enteritidis isolated from broiler carcasses. Braz. J. Microbiol..

[24] Cheah Y.-K., Learn-Han L., Noorzaleha A., Son R., Sabrina S., Jiun-Horng S., Chai-Hoon K. (2008). Characterization of multiple-antimicrobial-resistant Salmonella enterica Subsp. enterica isolated from indigenous vegetables and poultry in Malaysia. Lett. Appl. Microbiol..

[25] Thong K.L. (2010). Characterization of drug resistant Salmonella enterica Serotype Typhimurium by Antibiograms, Plasmids, Integrons, Resistance Genes and PFGE. J. Microbiol. Biotechnol..

[26] Khoo E., Roseliza R., Khoo L., Nafizah M., Saifu Nazri R., Hasnah Y., Norazariyah M., Rosnah Y., Rosna D., Siti Nor Hanani R. (2015). Antimicrobial resistance of Salmonella enterica serovar Typhimurium from various meats received in VRI. Malays. J. Vet. Res..

[27] Lamas A., Fernandez-No I.C., Miranda J.M., Vázquez B.I., Cepeda A., Franco C.M. (2016). Prevalence, molecular characterization and antimicrobial resistance of Salmonella serovars isolated from northwestern Spanish broiler flocks (2011–2015). Poult. Sci..

[28] Abdel-Maksoud M., Abdel-Khalek R., El-Gendy A., Gamal R.F., Abdelhady H.M., House B.L. (2015). Genetic characterisation of multidrug-resistant Salmonella enterica serotypes isolated from poultry in Cairo, Egypt. Afr. J. Lab. Med..

[29] Zhang L., Fu Y., Xiong Z., Ma Y., Wei Y., Qu X., Zhang H., Zhang J., Liao M. (2018). Highly Prevalent Multidrug-Resistant Salmonella From Chicken and Pork Meat at Retail Markets in Guangdong, China. Front. Microbiol..

[30] Eguale T. (2018). Non-typhoidal Salmonella serovars in poultry farms in central Ethiopia: Prevalence and antimicrobial resistance. BMC Vet. Res..

[31] Shang K., Wei B., Kang M. (2018). Distribution and dissemination of antimicrobial-resistant Salmonella in broiler farms with or without enrofloxacin use. BMC Vet. Res..

[32] Chashni E., Hassanzadeh S.H., Fard B. (2009). Characterization of the Salmonella Isolates from Backyard Chickens in North of Iran, by Serotyping, Multiplex PCR and Antibiotic Resistance Analysis. Arch. Razi Inst..

[33] Ghoddusi A., Fasaei B.N., Karimi V., Tamai I.A., Moulana Z., Salehi T.Z. (2015). Molecular identification of Salmonella Infantis isolated from backyard chickens and detection of their resistance genesby PCR. Iran. J. Vet. Res..

[34] Manning J., Gole V., Chousalkar K.K. (2015). Screening for Salmonella in backyard chickens. Prev. Vet. Med..

[35] Zhao X., Gao Y., Ye C., Yang L., Wang T., Chang W. (2016). Prevalence and Characteristics of Salmonella Isolated from Free-Range Chickens in Shandong Province, China. BioMed Res. Int..

[36] Jafari R., Ghorbanpour M., Jaideri A. (2007). An Investigation into Salmonella Infection Status in Backyard Chickens in Iran. Int. J. Poult. Sci..

[37] Geidam Y.A., Zakaria Z., Aziz S.A., Bejo S.K., Abu J., Omar S. (2012). High Prevalence of Multi-drug Resistant Bacteria in Selected Poultry Farms in Selangor, Malaysia. Asian J. Anim. Vet. Adv..

[38] Borges K.A., Furian T., Souza S., Salle C., Moraes H., Nascimento V.P.D. (2019). Antimicrobial Resistance and Molecular Characterization of Salmonella Enterica Serotypes Isolated from Poultry Sources in Brazil. Rev. Bras. Ciência Avícola.

[39] Rech D.V., Vaz C.S.L., Alves L., Coldebella A., Leão J.A., Rodrigues D.P., Back A. (2015). A temporal study of Salmonella enterica serotypes from broiler farms in Brazil. Poult. Sci..

[40] Esengupta S., Chattopadhyay M.K., Grossart H.-P. (2013). The multifaceted roles of antibiotics and antibiotic resistance in nature. Front. Microbiol..

[41] Lee J.H., Park K.S., Jeon J.H., Lee H.S. (2018). Antibiotic resistance in soil. Lancet Infect. Dis..

[42] Tyrrell C., Burgess C.M., Brennan F.P., Walsh F. (2019). Antibiotic resistance in grass and soil. Biochem. Soc. Trans..

[43] Almakki A., Jumas-Bilak E., Marchandin H., Licznar-Fajardo P. (2019). Antibiotic resistance in urban runoff. Sci. Total Environ..

[44] Sanganyado E., Gwenzi W. (2019). Antibiotic resistance in drinking water systems: Occurrence, removal, and human health risks. Sci. Total Environ..

[45] Shobrak M.Y., Abo-Amer A.E. (2015). Role of wild birds as carriers of multi-drug resistant Escherichia coli and Escherichia vulneris. Braz. J. Microbiol..

[46] Atterby C., Ramey A.M., Hall G.G., Järhult J., Börjesson S., Bonnedahl J. (2016). Increased prevalence of antibiotic-resistantE. coliin gulls sampled in Southcentral Alaska is associated with urban environments. Infect. Ecol. Epidemiol..

[47] Ramey A.M., Hernandez J., Tyrlöv V., Uher-Koch B.D., Schmutz J.A., Atterby C., Järhult J.D., Bonnedahl J. (2017). Antibiotic-Resistant Escherichia coli in Migratory Birds Inhabiting Remote Alaska. EcoHealth.

[48] Ahlstrom C.A., Bonnedahl J., Woksepp H., Hernandez J., Olsen B., Ramey A.M. (2018). Acquisition and dissemination of cephalosporin-resistant E. coli in migratory birds sampled at an Alaska landfill as inferred through genomic analysis. Sci. Rep..

[49] Singh R., Yadav A.S., Tripathi V., Singh R.P. (2013). Antimicrobial resistance profile of Salmonella present in poultry andpoultry environment in north India. Food Control.

[50] Musa I.W., Mansur M.S., Sa’Idu L., Mohammed B., Aliyu H.B. (2014). Poultry environment and farm practices influencing the isolation rate of multi-drug resistant Salmonella from water and poultry feed in Zaria, Nigeria. J. Appl. Biol. Biotechnol..

[51] Rossi F., Girardello R., Cury A.P., Di Gioia T.S.R., De Almeida J.N., Duarte A.J.D.S. (2017). Emergence of colistin resistance in the largest university hospital complex of São Paulo, Brazil, over five years. Braz. J. Infect. Dis..

[52] Rolain J.-M., Olaitan A.O., Information P.E.K.F.C. (2016). Plasmid-mediated colistin resistance: The final blow to colistin?. Int. J. Antimicrob. Agents.

[53] Liu Y.-Y., Wang Y., Walsh T.R., Yi L.-X., Zhang R., Spencer J., Doi Y., Tian G., Dong B., Huang X. (2016). Emergence of plasmid-mediated colistin resistance mechanism MCR-1 in animals and human beings in China: A microbiological and molecular biological study. Lancet Infect. Dis..

[54] Baron S., Hadjadj L., Rolain J.-M., Olaitan A.O. (2016). Molecular mechanisms of polymyxin resistance: Knowns and unknowns. Int. J. Antimicrob. Agents.

[55] Al-Tawfiq J.A., Laxminarayan R., Mendelson M. (2017). How should we respond to the emergence of plasmid-mediated colistin resistance in humans and animals?. Int. J. Infect. Dis..

[56] Huang X., Yu L., Chen X., Zhi C., Yao X., Liu Y., Wu S., Guo Z., Yi L., Zeng Z. (2017). High Prevalence of Colistin Resistance and mcr-1 Gene in Escherichia coli Isolated from Food Animals in China. Front. Microbiol..

[57] Hembach N., Schmid F., Alexander J., Hiller C., Rogall E.T., Schwartz T. (2017). Occurrence of the mcr-1 Colistin Resistance Gene and other Clinically Relevant Antibiotic Resistance Genes in Microbial Populations at Different Municipal Wastewater Treatment Plants in Germany. Front. Microbiol..

[58] Partridge S.R., Di Pilato V., Doi Y., Feldgarden M., Haft D.H., Klimke W., Kumar-Singh S., Liu J.-H., Malhotra-Kumar S., Prasad A. (2018). Proposal for assignment of allele numbers for mobile colistin resistance (mcr) genes. J. Antimicrob. Chemother..

[59] Wang X., Wang Y., Zhou Y., Li J., Yin W., Wang S., Zhang S., Shen J., Shen Z., Wang Y. (2018). Emergence of a novel mobile colistin resistance gene, mcr-8, in NDM-producing Klebsiella pneumoniae. Emerg. Microbes Infect..

[60] Wang X., Wang Y., Zhou Y., Wang Z., Wang Y., Zhang S., Shen Z. (2019). Emergence of Colistin Resistance Gene mcr-8 and Its Variant in Raoultella ornithinolytica. Front. Microbiol..

[61] Stoesser N., Mathers A.J., E Moore C., Day N.P.J., Crook D.W. (2016). Colistin resistance gene mcr-1 and pHNSHP45 plasmid in human isolates of Escherichia coli and Klebsiella pneumoniae. Lancet Infect. Dis..

[62] Ström G., Börjesson S., Sokerya S., Sothyra T., Magnusson U. (2019). Detection of mcr-Mediated Colistin Resistance in Escherichia coli Isolates from Pigs in Small-Scale Farms in Cambodia. Antimicrob. Agents Chemother..

[63] Yassin A.K., Zhang J., Wang J., Chen L., Kelly P., Butaye P., Lu G., Gong J., Li M., Wei L. (2017). Identification and characterization of mcr mediated colistin resistance in extraintestinal Escherichia coli from poultry and livestock in China. FEMS Microbiol. Lett..

[64] Li J., Hulth A., E Nilsson L., Börjesson S., Chen B., Bi Z., Wang Y., Schwarz S., Wu C. (2018). Occurrence of the mobile colistin resistance gene mcr-3 in Escherichia coli from household pigs in rural areas. J. Antimicrob. Chemother..

[65] Kusumoto M., Ogura Y., Gotoh Y., Iwata T., Hayashi T., Akiba M. (2016). Colistin-resistant mcr-1 –positive pathogenic Escherichia coli in swine, Japan, 2007−2014. Emerg. Infect. Dis..

[66] Ohsaki Y., Hayashi W., Saito S., Osaka S., Taniguchi Y., Koide S., Kawamura K., Nagano Y., Arakawa Y., Nagano N. (2017). First Detection of an Escherichia coli Strain Harboring the mcr-1 Gene in Retail Domestic Chicken Meat in Japan. Jpn. J. Infect. Dis..

[67] Olaitan A.O., Thongmalayvong B., Akkhavong K., Somphavong S., Paboriboune P., Khounsy S., Morand S., Rolain J.-M. (2015). Clonal transmission of a colistin-resistant Escherichia coli from a domesticated pig to a human in Laos: Table 1. J. Antimicrob. Chemother..

[68] Joshi P.R., Thummeepak R., Paudel S., Acharya M., Pradhan S., Banjara M.R., Leungtongkam U., Sitthisak S. (2019). Molecular Characterization of Colistin-Resistant Escherichia coli Isolated from Chickens: First Report from Nepal. Microb. Drug Resist..

[69] Mohsin M., Raza S., Roschanski N., Schaufler K., Guenther S. (2016). First description of plasmid-mediated colistin-resistant extended-spectrum β-lactamase-producing Escherichia coli in a wild migratory bird from Asia. Int. J. Antimicrob. Agents.

[70] Azam M., Ehsan I., Rahman S.-U., Saleemi M.K., Javed M.R., Mohsin M. (2017). Detection of the colistin resistance gene mcr-1 in avian pathogenic Escherichia coli in Pakistan. J. Glob. Antimicrob. Resist..

[71] Ghafur A., Shankar C., Soundari P.G., Venkatesan M., A Thirunarayan M., Mani D., Veeraraghavan B. (2019). Detection of chromosomal and plasmid-mediated mechanisms of colistin resistance in Escherichia coli and Klebsiella pneumoniae from Indian food samples. J. Glob. Antimicrob. Resist..

[72] Eiamphungporn W., Yainoy S., Jumderm C., Tan-Arsuwongkul R., Tiengrim S., Thamlikitkul V. (2018). Prevalence of the colistin resistance gene mcr-1 in colistin-resistant Escherichia coli and Klebsiella pneumoniae isolated from humans in Thailand. J. Glob. Antimicrob. Resist..

[73] Malhotra-Kumar S., Xavier B.B., Das A.J., Lammens C., Hoang H.T.T., Pham N.T., Goossens H. (2016). Colistin-resistant Escherichia coli harbouring mcr-1 isolated from food animals in Hanoi, Vietnam. Lancet Infect. Dis..

[74] Yamaguchi T., Kawahara R., Harada K., Teruya S., Nakayama T., Motooka D., Nakamura S., Nguyen P.D., Kumeda Y., Van Dang C. (2018). The presence of colistin resistance gene mcr-1 and -3 in ESBL producing Escherichia coli isolated from food in Ho Chi Minh City, Vietnam. FEMS Microbiol. Lett..

[75] Jajere S.M., Hassan L., Aziz S.A., Zakaria Z., Abu J., Nordin F., Faiz N.M. (2019). Salmonella in native “village” chickens (Gallus domesticus): Prevalence and risk factors from farms in South-Central Peninsular Malaysia. Poult. Sci..

[76] Clinical and Laboratory Standards Institute (2018). Performance Standards for Antimicrobial Disk and Dilution Susceptibility Tests for Bacteria Isolated from Animals.

[77] Matuschek E., Åhman J., Webster C., Kahlmeter G. (2018). Antimicrobial susceptibility testing of colistin—evaluation of seven commercial MIC products against standard broth microdilution for Escherichia coli, Klebsiella pneumoniae, Pseudomonas aeruginosa, and Acinetobacter spp.. Clin. Microbiol. Infect..

[78] World Health Organization (2011). Critically Important Antimicrobials for Human Medicine.

[79] Magiorakos A.-P., Srinivasan A., Carey R., Carmeli Y., Falagas M., Giske C., Harbarth S., Hindler J., Kahlmeter G., Olsson-Liljequist B. (2012). Multidrug-resistant, extensively drug-resistant and pandrug-resistant bacteria: An international expert proposal for interim standard definitions for acquired resistance. Clin. Microbiol. Infect..

[80] Amoako D.G., Somboro A.M., Abia A.L.K., Molechan C., Perrett K., Bester L.A., Essack S.Y. (2020). Antibiotic Resistance in Staphylococcus aureus from Poultry and Poultry Products in uMgungundlovu District, South Africa, Using the “Farm to Fork” Approach. Microb. Drug Resist..

[81] Krumperman P.H. (1983). Multiple antibiotic resistance indexing of Escherichia coli to identify high-risk sources of fecal contamination of foods. Appl. Environ. Microbiol..

